# Cardiac action of the first G protein biased small molecule apelin agonist

**DOI:** 10.1016/j.bcp.2016.07.018

**Published:** 2016-09-15

**Authors:** Cai Read, Christopher M. Fitzpatrick, Peiran Yang, Rhoda E. Kuc, Janet J. Maguire, Robert C. Glen, Richard E. Foster, Anthony P. Davenport

**Affiliations:** a Experimental Medicine and Immunotherapeutics, Addenbrooke’s Hospital, University of Cambridge, UK; b School of Chemistry and Astbury Centre for Structural Biology, University of Leeds, UK; c Centre for Molecular Informatics, Department of Chemistry, University of Cambridge, UK; d Biomolecular Medicine, Department of Surgery and Cancer, Imperial College London, London, UK

**Keywords:** Apelin, Biased agonism, G protein coupled receptor, Cardiovascular, *In vivo*

## Abstract

Apelin peptide analogues displaying bias towards G protein signalling pathways have beneficial cardiovascular actions compared with the native peptide in humans *in vivo*. Our aim was to determine whether small molecule agonists could retain G protein bias. We have identified a biased small molecule, CMF-019, and characterised it *in vitro* and *in vivo*.

In competition radioligand binding experiments in heart homogenates, CMF-019 bound to the human, rat and mouse apelin receptor with high affinity (pK_i_ = 8.58 ± 0.04, 8.49 ± 0.04 and 8.71 ± 0.06 respectively). In cell-based functional assays, whereas, CMF-019 showed similar potency for the G_αi_ pathway to the endogenous agonist [Pyr^1^]apelin-13 (pD_2_ = 10.00 ± 0.13 vs 9.34 ± 0.15), in β-arrestin and internalisation assays it was less potent (pD_2_ = 6.65 ± 0.15 vs 8.65 ± 0.10 and pD_2_ = 6.16 ± 0.21 vs 9.28 ± 0.10 respectively). Analysis of these data demonstrated a bias of ∼400 for the G_αi_ over the β-arrestin pathway and ∼6000 over receptor internalisation. CMF-019 was tested for *in vivo* activity using intravenous injections into anaesthetised male Sprague–Dawley rats fitted with a pressure-volume catheter in the left ventricle. CMF-019 caused a significant increase in cardiac contractility of 606 ± 112 mmHg/s (p < 0.001) at 500 nmol. CMF-019 is the first biased small molecule identified at the apelin receptor and increases cardiac contractility *in vivo*. We have demonstrated that G_αi_ over β-arrestin/internalisation bias can be retained in a non-peptide analogue and predict that such bias will have the therapeutic benefit following chronic use. CMF-019 is suitable as a tool compound and provides the basis for design of biased agonists with improved pharmacokinetics for treatment of cardiovascular conditions such as pulmonary arterial hypertension.

## Graphical abstract

**Figure:**
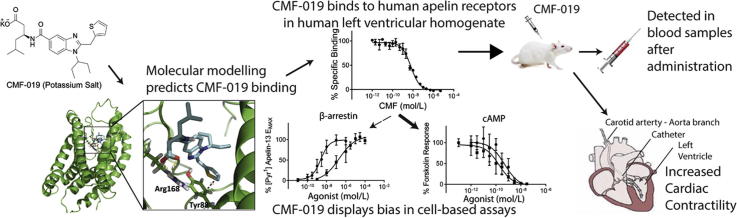

## 1 Introduction

The apelin receptor is a class A G protein-coupled receptor first cloned in 1993 [1] and was deorphanised in 1998 when its cognate ligand, apelin, was identified from bovine stomach extracts [2]. The predominant isoform in the human cardiovascular system is [Pyr^1^]apelin-13 [3]. Infusion of apelin leads to vasodilatation, in humans *in vitro* [3] and *in vivo* [4] and in rodents *in vivo* [5], as well as cardiac inotropy *in vitro* [3], [6], [7] and *in vivo* in rats [8], [9], [10], mice [11] and humans [12] without hypertrophy.

These properties of apelin signalling have made it of particular interest as a potential therapeutic for a number of diseases such as pulmonary arterial hypertension (PAH) and heart failure (HF) [13]. Moreover, there is significant evidence to suggest that whilst the apelin peptide is downregulated in both PAH [14], [15], [16] and HF [17], [18], [19], expression of the receptor remains unchanged and responsive to apelin [15], [20], [21]. This provides an opportunity to replace the downregulated endogenous agonist in a therapeutic setting. Previous work has shown that infusion of apelin is beneficial in a number of disease models of PAH [22] and HF [8], [9], [10], [23], [24], [25], but long term therapeutic efficacy is limited by a short half-life of a few minutes. Oral therapy for these chronic diseases would necessitate a compound with longer half-life. Furthermore, upon activation of the receptor, bound apelin is rapidly internalised through the β-arrestin pathway with the rate of recycling back to the plasma membrane highly dependent on the ligand [26], [27]. Therefore, agonist induced desensitisation may limit clinical efficacy.

We hypothesise that a G protein biased small molecule apelin agonist could provide a solution to this limitation. We have previously designed apelin peptide analogues that are G protein biased and this strategy resulted in an improved duration of action and increased efficacy [4]. Alteration of a specific serine residue (Ser^348^) in the apelin receptor has been shown to abolish the G protein receptor kinase/β-arrestin pathway signalling whilst preserving signalling through the G protein pathway. It has also been reported that removal of the C-terminal phenylalanine of apelin-17 can induce bias towards G protein signalling [28]. These reports suggest that the apelin receptor is tractable to biased signalling [29]. Here we identify CMF-019, a small molecule, which binds to the human apelin receptor with high affinity, exerts a biased response through the G protein pathway and mimics the beneficial cardiovascular actions of apelin in rodents.

## 2 Methods

### 2.1 Materials

Chemicals were obtained from Sigma–Aldrich Co. Ltd (Poole, UK) unless otherwise stated. [Pyr^1^]apelin-13 (Glp-RPRLSHKGPMPF) was from Severn Biotech (Kidderminster, UK), [Glp^65^,Nle^75^,Tyr^77^][125^I^]apelin-13, was from Perkin Elmer (MA, USA). CMF-019 was synthesised in the School of Chemistry, University of Leeds. Human tissues were obtained with informed consent from the Papworth Hospital Research Tissue Bank (08/H0304/56) and ethical approval (05/Q104/142) and conformed to the principles outlined in the declaration of Helsinki. Animal experiments were performed in accordance with guidelines from the local ethics committee (University of Cambridge) and the Home Office (UK) under the Scientific Procedures Act (1986).

### 2.2 Homology modelling of the apelin receptor/apelin-13 and ligand receptor docking of CMF-019

A homology model was constructed from the 2.5 Å resolution crystal structure of the human CXCR4 chemokine receptor. MODELLER9v8 was used to generate homology models of apelin which were subsequently refined using molecular dynamics. A more detailed description of the procedure is given in Brame et al. (2015) [4]. Water and ions were removed in preparation for docking studies. The model of CMF-019 (carboxylic acid ionised) was constructed using Sybyl 7.3 and energy minimised *in vacuo* using the TriposFF and Gasteiger-Huckel charges. Docking was performed using the programme GOLD (CCDC 2015) [30], [31].

It was hypothesised that the agonist, CMF-019, occupied a site in close proximity to the site occupied by apelin-13 within the pocket formed by the seven transmembrane helices of the receptor. However, initial docking of CMF-019 into this long pocket formed from the receptor model resulted in a number of possible solutions. To constrain the system, we utilised the previously determined homology model of bound apelin-13 [4], selecting the region containing the serine-histidine-lysine (SHK) sequence of apelin-13 as the most promising region for binding of CMF-019 (which is adjacent to the important residue Tyr^88^ identified from mutagenesis studies). A receptor cavity composed of residues up to 12 Å from the SHK region of apelin-13 was used for docking. The ChemPLP scoring function was used with standard GOLD settings.

### 2.3 Synthesis of CMF-019

**Fig. 1:**
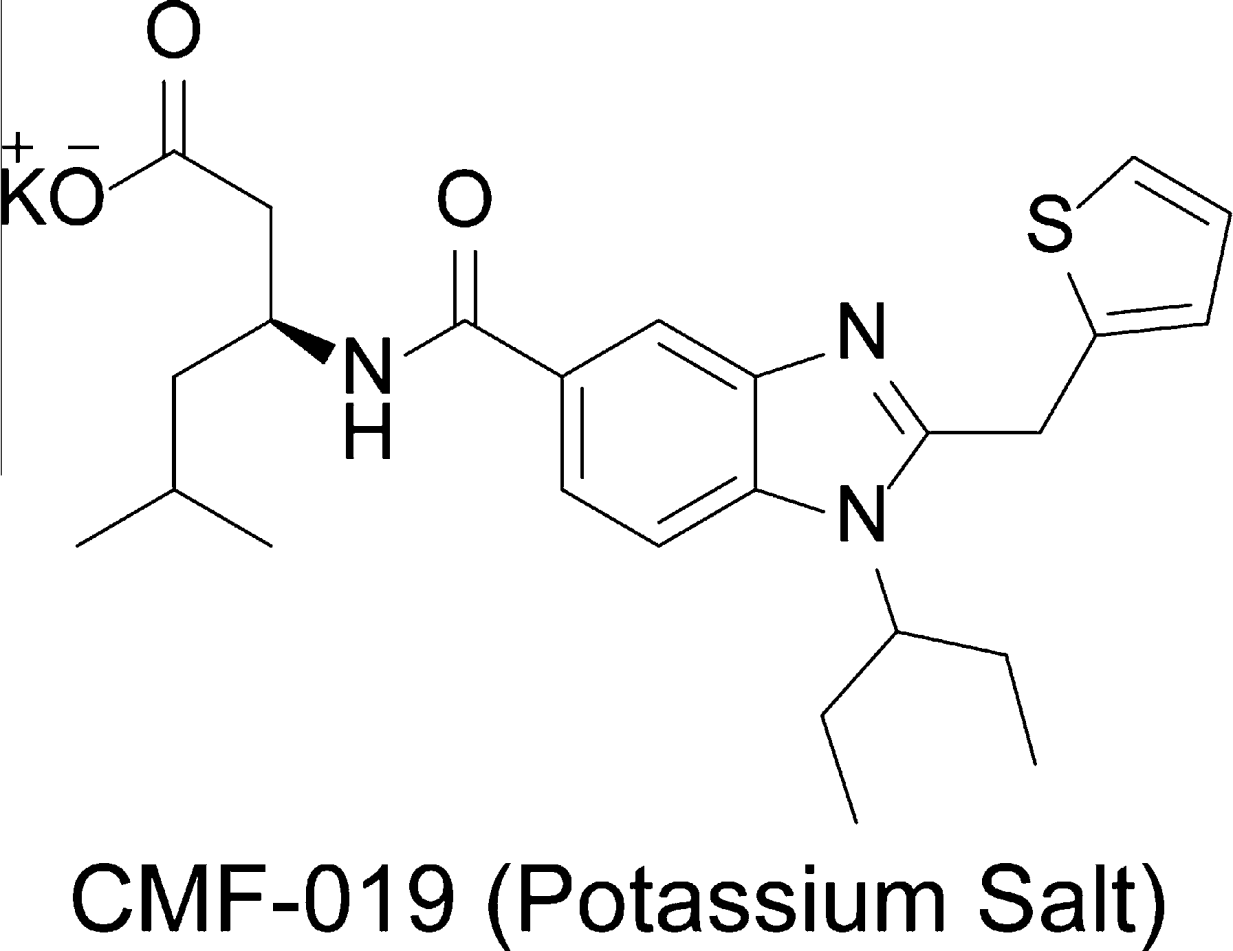
Structure of the potassium salt of CMF-019.

**Fig. 2:**
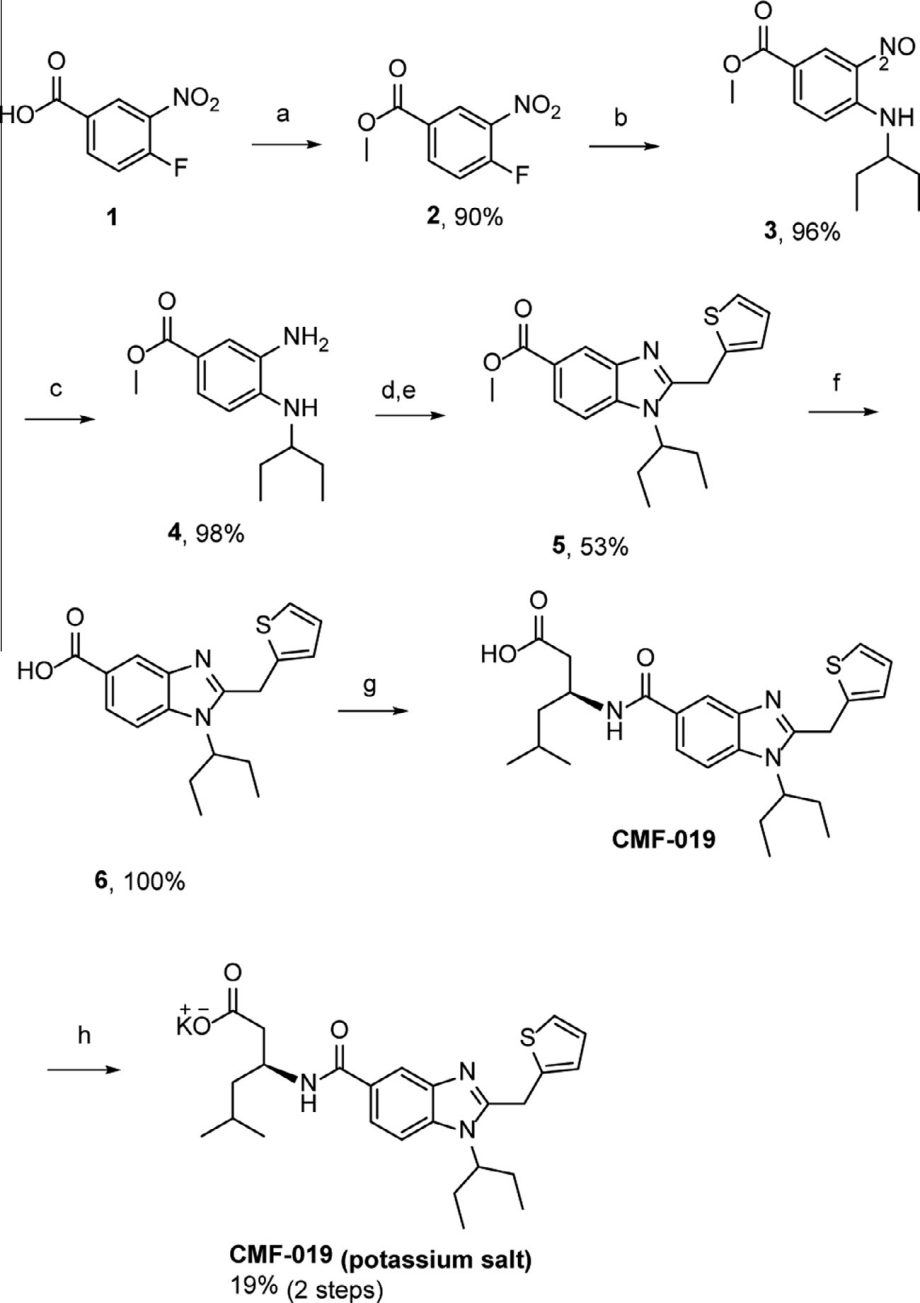
Synthetic pathway for the production of the potassium salt of CMF-019. Intermediates: (1) 4-fluoro-3-nitrobenzoic acid, (2) Methyl-4-fluoro-3-nitrobenzoate, (3) 3-(3-aminopentane)-4-nitrobenzylmethanoate, (4) Methyl-3-amino-4-(pentan-3-ylamino)benzoate, (5) Methyl-1-(pentan-3-yl)-2-(thiophen-2-ylmethyl)-1*H*-benzo[d]imidazole-5-carboxylate, (6) 1-(Pentan-3-yl)-2-(thiophen-2-ylmethyl)-1*H*-benzo[d]imidazole-5-carboxylic acid. Reagents and conditions: (a) SOCl_2_, MeOH, (b) K_2_CO_3_, 3-aminopentane, DMF, (c) 5% Pd/C, H_2_ (15 bar), MeOH, (d) 2-thiophene acetic acid, SOCl_2_, DCM, (e) acetic acid, 140 °C (microwave), (f) NaOH, MeOH, 110 °C (microwave), (g) (i) HCTU, DIPEA, DMF, (ii) NaOH, MeOH, (h) KOH, 1,4-dioxane.

CMF-019 ((*S*)-3-[1-(1-Ethyl-propyl)-2-thiophen-2-ylmethyl-1H-benzoimidazole-5-carbonyl]-amino-5-methyl-hexanoic acid) has previously been prepared (compound number 107 from patent US20140094450, 2014) via a minor modification of the literature procedure (Fig. 2) [32]. For *in vivo* studies we used the potassium salt of the compound (Fig. 1) dissolved in saline at pH9 as it showed better solubility than the parent compound. Thermodynamic solubility (potassium salt) and an *in vitro* murine microsomal stability of CMF-019 were performed at Cyprotex (Macclesfield, UK).

### 2.4 Competition radioligand binding

Initial binding assays were performed by Cerep (Celle L’Evescault, France) on Chinese Hamster Ovary (CHO-K1) cells expressing the human apelin receptor. Further experiments were performed on human homogenised left ventricle (HLV, 1.5 mg/ml) and rat and mouse whole heart homogenates to determine any species variability prior to *in vivo* experiments. Competition binding experiments were carried out using [Glp^65^,Nle^75^,Tyr^77^][^125^I]apelin-13 (0.1 nmol/L) as described previously [4], with unlabelled CMF-019 (2 pmol/L-10 μmol/L) as the competitive agonist. Binding in the presence of 2 μmol/L [Pyr^1^]apelin-13 was considered non-specific. Experiments were performed in triplicate and data analysed using GraphPad Prism 6 (GraphPad Software, Inc. La Jolla, USA). Binding affinities were calculated by the Cheng-Prusoff methodology using measured IC_50_ values.

### 2.5 Cell-based assays

β-Arrestin recruitment, β-arrestin mediated internalisation and cAMP assays were performed according to the manufacturer’s instructions (DiscoverX®, Fremont, CA) and as described previously [4]. Agonist responses, measured in relative light units, were expressed as a percentage relative to the E_MAX_ of [Pyr^1^]apelin-13 for β-arrestin and internalisation experiments and as a percentage relative to the forskolin response for cAMP experiments. Data were fitted to a four parameter model using GraphPad Prism 6 and the pD_2_ (−log_10_ of the EC_50_ (the concentration producing 50% of the maximum response)) and maximum response (E_MAX_) values were calculated and compared.

### 2.6 Rat heart catheterisation and jugular vein cannulation for *in vivo* assessment of CMF-019

Male Sprague–Dawley rats (273 ± 6 g; Charles River Laboratories, Margate, UK) were induced to anaesthesia with inhalation of 3% isoflurane and then maintained under 1.5% isoflurane via a face mask. Temperature was monitored throughout the surgical procedure using a rectal probe. Prior to the start of surgery, it was ensured that pain reflexes had ceased by use of a hind-paw pinch test. The surgery was completed as previously described [33]. In brief, the right external jugular vein was cannulated, flushed with heparin solution (2%, 0.9% saline, pH5, Macopharma) and the right common carotid artery located. The catheter (Millar Inc., SPR-869) was calibrated using the MPVS Ultra system (ADIstruments) before being inserted into the carotid and advanced to the left ventricle. Once a stable pressure–volume loop could be observed, the catheter was left in place for ten minutes. Three cumulative bolus doses of CMF-019 (50–5000 nmol, 0.5 mL, 0.9% saline, pH9, Macopharma, n = 7–9) were then administered intravenously, followed by a saline flush (0.9%, 0.1 mL, pH5) via the cannula at ten minute intervals, so that a stable baseline was reached before the next injection. The effects on pressure and volume were measured and the heart rate monitored to determine and maintain a suitable depth of anaesthesia. Control animals were injected with saline (0.9%, 0.5 mL, pH9) and a flush of equal volume (0.9%, 0.1 mL, pH5) to distinguish the drug effects from any volume effects (n = 3–5). The effects of [Pyr^1^]apelin-13 (50, 400 nmol, 0.5 mL, 0.9% saline, pH5, Macopharma, n = 8) were studied for comparison. Animals were randomly chosen to receive CMF-019, saline or [Pyr^1^]apelin-13 injection. Data were acquired using the MPVS Ultra system (ADIstruments) and analysed using LabChart 8 (ADIstruments). Values for the maximal change in left ventricular systolic pressure (LVSP), stroke volume, cardiac output, contractility (dP/dt_MAX_) and lusitropy (dP/dt_MIN_) from baseline were calculated from the raw data and compared. Following completion of the measurements the animal was euthanised by exsanguination under high flow isoflurane.

### 2.7 Analysis of plasma samples by mass spectrometry

Analysis of plasma samples was performed by Peakdale Molecular (Chapel-en-la-Frith, UK). End-point blood samples were taken from rats (n = 6) following completion of three cumulative doses of CMF-019 (as described before), approximately 10 min after the last intravenous administration. The blood was collected in heparin-coated vials and spun at low speed (2000 g, 5 min), the plasma supernatant was removed and frozen. Quantitative analysis was performed using LC-MS/MS with metaprolol as an internal standard and CMF-019 to quantify CMF-019 in the samples.

### 2.8 Computational assessment of oral drug properties

CMF-019 was assessed for drug-like properties using FAF-Drugs3 [34]. This compared the properties of CMF-019 to a library of oral drugs and predicted its physiochemical properties according to Lipinski’s and Verber’s rules. In addition, CMF-019 was analysed using a principal component analysis (PCA) of 15 key physiochemical properties and compared to the properties of oral drugs in current use, extracted as sublibraries from the databases, eDrugs and DrugBank.

### 2.9 Statistical analysis

All data are expressed as mean ± SEM values. Binding and cell-based experiments were performed in triplicate. For cell based assays, n-values are given as the number of replicates/number of experiments. Bias calculations were performed, as previously described [35], to obtain values for relative effectiveness of CMF-019 compared to [Pyr^1^]apelin-13 within each cell-based assay and bias factors calculated to compare the relative activities of the agonists between different pathways. For the *in vivo* study, values for the cardiovascular parameters measured in saline and CMF-019 treated animals were compared using two-tailed student’s *t*-test (GraphPad Prism 6). Statistical significance was taken as 5%.

## 3 Results

### 3.1 Computational docking of CMF-019

**Fig. 3:**
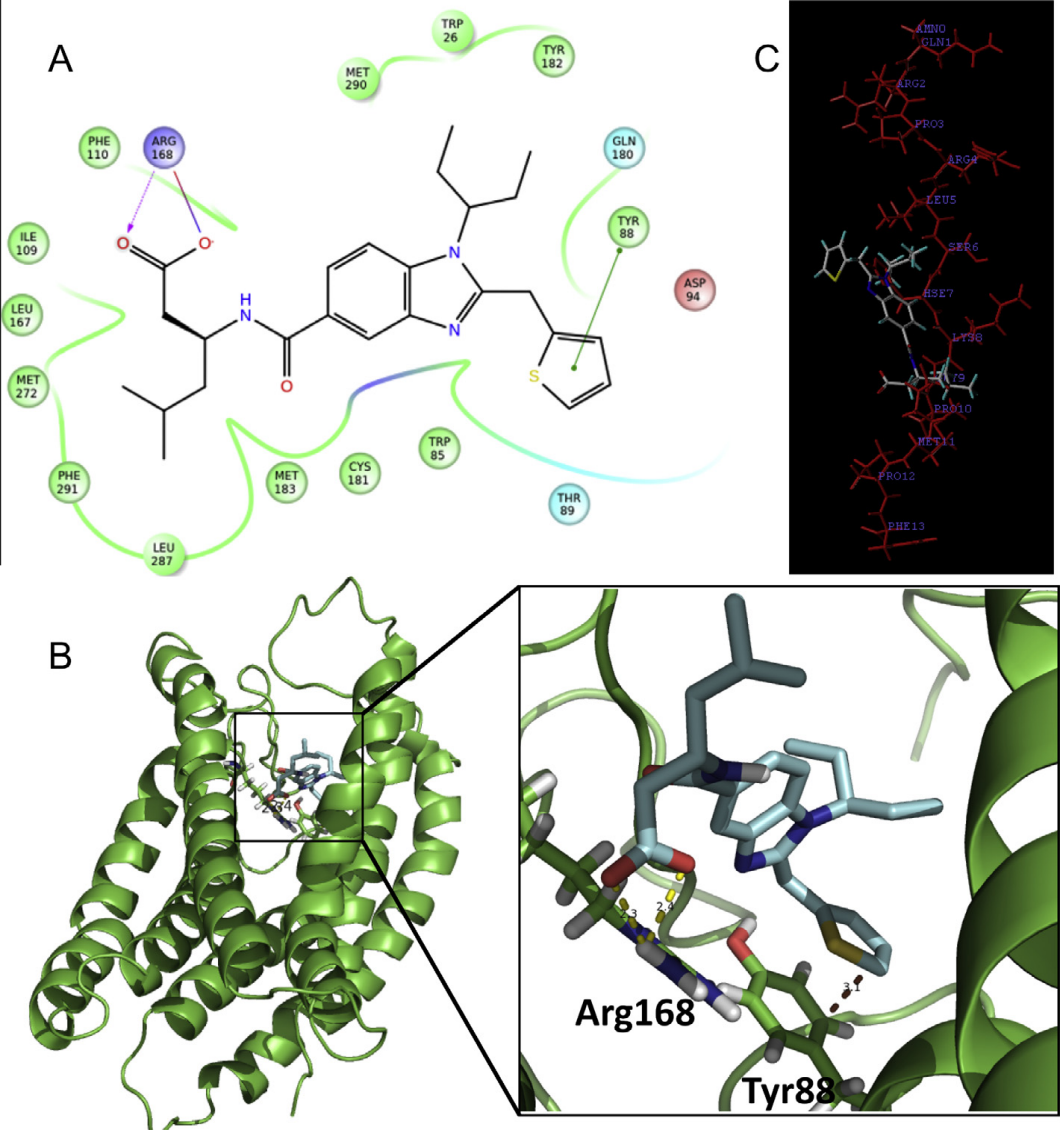
Computational docking of CMF-019. A 2D interaction map (A) showing the binding site and interactions between CMF-019 and the APJ receptor. The key interactions are π-stacking (thiophene to Tyr^88^, green line) and ionic (carboxylate to Arg^168^, purple lines) bonds. CMF-019 (pale blue sticks) docked via GOLD into the APJ receptor (green) (B). The key binding interactions between the ligand and APJ receptor (at Arg^168^ and Tyr^88^) are highlighted (dotted yellow lines) with intermolecular distances shown. The favoured overlay of apelin 13 (red lines) with CMF-019 (grey lines) (C) places CMF-019 at the SHK region, suggesting that the SHK sequence of apelin-13 is the most promising region for binding of CMF-019 to the APJ receptor. (For interpretation of the references to colour in this figure legend, the reader is referred to the web version of this article.)

CMF-019 bound into a mainly hydrophobic cavity on the apelin receptor near the SHK region of bound apelin-13. The carboxylic acid of CMF-019 was predicted to form a strong hydrogen bond (2.3 Å) with the side-chain of Arg^168^. The thiophene, which is important for maintenance of good potency, formed a π-stacking interaction with residue Tyr^88^. A ligand/protein plot of these close interactions between CMF-019 and the apelin receptor was generated (Fig. 3A), as well as a close up view of the ligand receptor interactions (Fig. 3B). To compare the similarity in the expected binding regions of CMF-019 and the predicted apelin-13 docked structure, they were overlaid (Fig. 3C).

### 3.2 CMF-019 binds to the human apelin receptor with high affinity

**Fig. 4:**
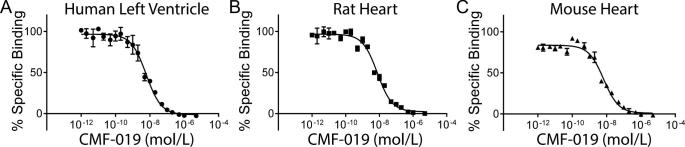
Competition binding experiments in heart tissue homogenates. The specific binding of CMF-019 to human left ventricular homogenate (A, ●), rat whole heart homogenate (B, ■) and mouse whole heart homogenate (C, ▴).

CMF-019 bound to the human apelin receptor expressed in CHO cells with nanomolar affinity (pK_i_ = 7.61 ± 0.14). In human left ventricle, CMF-019 showed monophasic competition and a ten-fold higher binding affinity to the native apelin receptor (pK_i_ = 8.58 ± 0.04, Fig. 4A). Similar binding was observed in rat and mouse heart (pK_i_ = 8.49 ± 0.04, Fig. 4B; pK_i_ = 8.71 ± 0.06, Fig. 4C respectively).

### 3.3 CMF-019 displays G protein bias

**Fig. 5:**
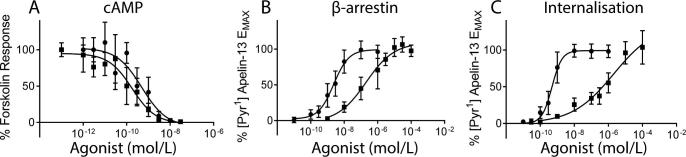
Pathway selectivity of CMF-019 determined from cell-based assays. The concentration response curves for [Pyr^1^]apelin-13 (●) and CMF-019 (■) in cell-based cAMP (A, ●n = 8/4, ■n = 11/4), β-arrestin (B, ●n = 12/4, ■n = 13/4) and internalisation (C, ●n = 6/2, ■n = 6/2) assays. Bias was calculated as described previously [35], relative effectiveness (RE) and bias factors (BF) are shown in Table 1, Table 2 respectively.

CMF-019 induced β-arrestin recruitment (Fig. 5B) to the apelin receptor (pD_2_ = 6.65 ± 0.15, n = 13/4) and subsequent receptor internalisation (Fig. 5C) **(**pD_2_ = 6.16 ± 0.21, n = 6/2) but was less potent than [Pyr^1^]apelin-13 (pD_2_ = 8.65 ± 0.10, n = 12/4; pD_2_ = 9.28 ± 0.10, n = 6/2 respectively). In contrast, CMF-019 potently inhibited G_αi_-mediated cAMP production (Fig. 5A) (pD_2_ = 10.00 ± 0.13, n = 11/4), comparable to [Pyr^1^]apelin-13 (pD_2_ = 9.34 ± 0.15, n = 8/4). In all assays, E_Max_ values for CMF-019 were similar to those obtained for [Pyr^1^]apelin-13 suggesting that the compound acted as a full agonist at the apelin receptor.

To quantify the degree to which CMF-019 displayed bias compared to the reference agonist [Pyr^1^]apelin-13, relative effectiveness (RE) and bias factors (BF) were calculated as described previously [35] and are shown in Table 1, Table 2 respectively.

### 3.4 CMF-019 is a positive inotrope *in vivo*

**Fig. 6:**
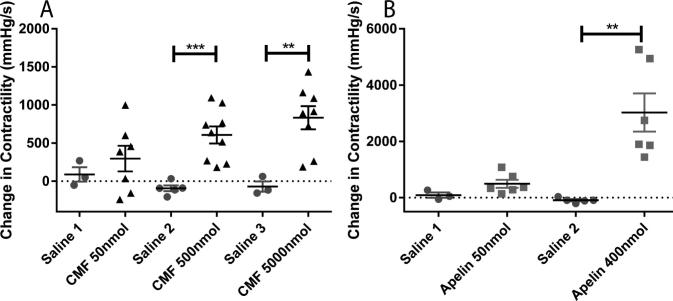
Cardiac contractility of CMF-019 *in vivo*. Dose dependent increases in left ventricular contractility in anaesthetised rats to (A) intravenous CMF-019 potassium salt (▴, n = 7–9) and (B) [Pyr^1^]apelin-13 (apelin, ■, n = 8) compared to saline (●, n = 3–5) control. Each dose was compared by a Student’s *t*-test to its corresponding saline control as doses were administered cumulatively (^*^p < 0.05, ^**^p < 0.01, ^***^P < 0.001).

**Fig. 7:**
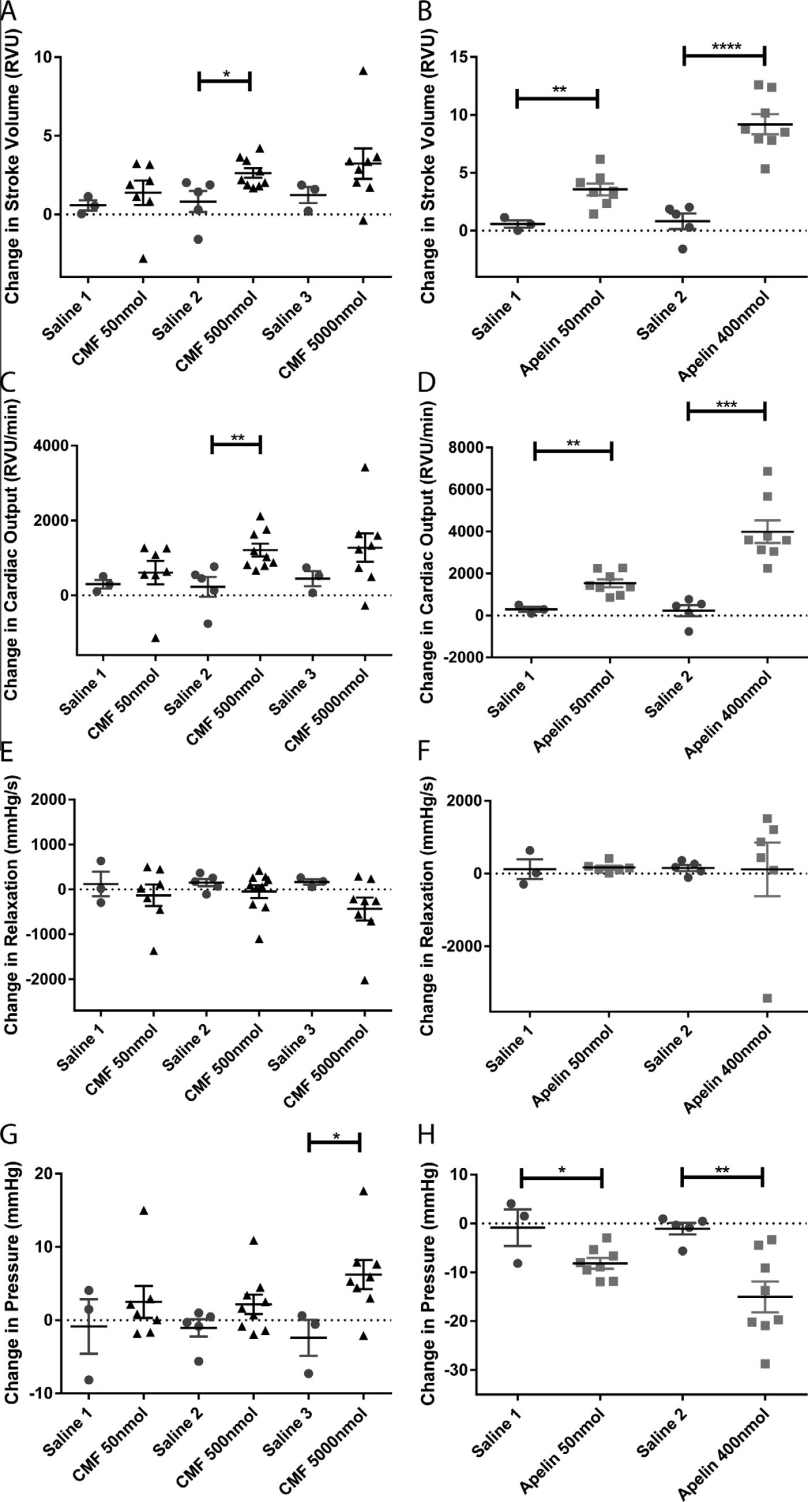
Cardiovascular actions of CMF-019 *in vivo*. Graphs showing changes in stroke volume (A-B), cardiac output (C-D), relaxation (dp/dt_MIN_) (E-F) and left ventricular systolic pressure (LVSP) (G-H) for CMF-019 potassium salt (▴, n = 7–9, A,C,E,G) and [Pyr^1^]apelin-13 (apelin, ■, n = 8, B,D,F,H) compared to saline (●, n = 3–5, A-H) when injected intravenously into anaesthetised rats. Each dose was compared by Student’s *t*-test to its corresponding saline control as doses were administered cumulatively (^*^p < 0.05, ^**^p < 0.01, ^***^P < 0.001, ^****^P < 0.0001).

Injection of CMF-019 caused a dose dependent increase in the dP/dt_MAX,_ a measure of cardiac contractility, with significance at 500 nmol of 606 ± 112 mmHg/s (p < 0.001) and at 5000 nmol of 833 ± 152 mmHg/s (p < 0.01) (Fig. 6A). [Pyr^1^]apelin-13 also caused a dose dependent increase with a significant response at 400 nmol of 3025 ± 680 mmHg/s (P < 0.01) (Fig. 6B). [Pyr^1^]apelin-13 dose dependently caused a significant increase in stroke volume and cardiac output up to a response at 400 nmol of 9.18 ± 0.86RVU (P < 0.0001) and 3989 ± 537RVU/min (P < 0.001) respectively. CMF-019 similarly increased these parameters with smaller responses detected. Changes at 500 nmol of 2.62 ± 0.31RVU (P < 0.05) and 1210 ± 170RVU/min (P < 0.01) respectively were significant (Fig. 7A,C). Neither [Pyr^1^]apelin-13 nor CMF-019 altered the dP/dt_MIN_, a measure of lusitropy (Fig. 7E-F). [Pyr^1^]apelin-13 caused a significant dose dependent drop in the LVSP with a maximal drop of 15.7 ± 1.8 mmHg detected at 400 nmol (P < 0.01) (Fig. 7G), however, CMF-019 resulted in a dose dependent increase in pressure with a significant increase of 6.22 ± 1.98 mmHg at 5000 nmol (P < 0.05) (Fig. 7H). A limitation of this study was the relatively low solubility of CMF-019 (potassium salt) in saline (2.09 mg/mL at pH7.4) that did not allow higher doses to be infused. No adverse effects were observed at any of the concentrations administered.

### 3.5 CMF-019 is present in rat plasma samples

CMF-019 had a half-life of 38 min in the *in vitro* microsomal stability experiment. Analysis of the plasma samples taken from rats treated with CMF-019 (potassium salt) demonstrated a mean plasma concentration of 25.4 ± 2.5 μM (heparinised samples, n = 6).

### 3.6 CMF-019 demonstrates good physiochemical properties for an oral drug

**Fig. 8:**
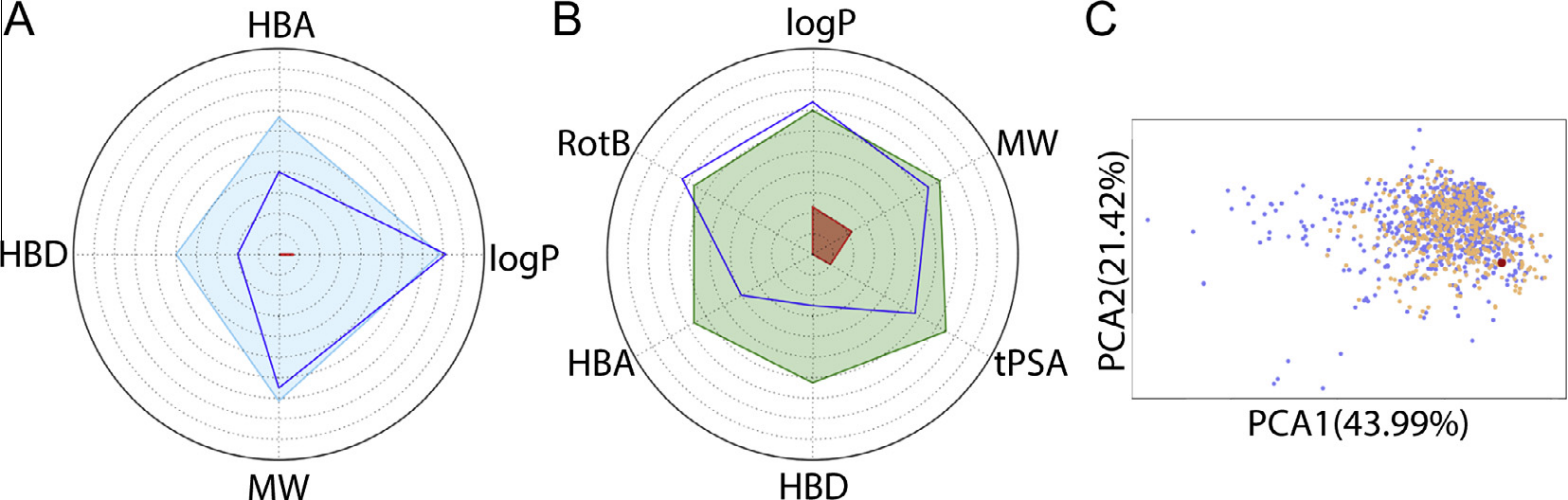
Assessment of drug-like properties of CMF-019 using FAF-Drugs3 [34]. (A) The properties of CMF-019 (HBD; H-bond donors; HBA, H-bond acceptors; logP, Octanol-water partition coefficient; MW, molecular weight) are shown as a dark blue line. A compound library of oral drugs was also assessed for these characteristics to illustrate the desirable physiochemical properties for a drug-like molecule according to Lipinski’s rules (light blue line). The red area denotes where it was not possible to have a molecule with those properties, or where there was a lack of data in the library. (B) An oral absorption estimation for CMF-019 (dark blue line) (HBD; H-bond donors; HBA, H-bond acceptors; RotB, rotatable bonds, logP, Octanol-water partition coefficient; MW, molecular weight; tPSA, topological polar surface area). A compound library of oral drugs was also assessed for these characteristics to illustrate the desirable physiochemical properties for a drug-like molecule according to Verber’s rules (dark green line). The red area denotes where it was not possible to have a molecule with those properties, or where there was a lack of data in the library. (C) An oral property space obtained by applying a Principal Component Analysis (PCA) of 15 physicochemical descriptors of CMF-019 (shown as the red dot), compared to properties of oral drugs in current use, extracted as sublibraries from the databases, eDrugs (blue) and DrugBank (orange). (For interpretation of the references to colour in this figure legend, the reader is referred to the web version of this article.)

Analysis of CMF-019 using FAF-Drugs3 [34] demonstrated a good physiochemical profile when compared to an oral drug library for Lipinski’s rules (Fig. 8A), Verber’s rules (Fig. 8B) and in a PCA analysis of 15 physiochemical descriptors (Fig. 8C). CMF-019 had only one Lipinski’s Rule-of-Five violation which was for logP (the octanol-water partition coefficient).

## 4 Discussion

### 4.1 CMF-019 is the first evaluated biased small molecule agonist at the apelin receptor

We have characterised and quantified the pharmacological properties of a small molecule, CMF-019, predicted to bind to the apelin receptor in a hydrophobic cavity near the SHK region of bound apelin-13 in computational models. CMF-019 demonstrated high affinity binding to native human, rat and mouse cardiac apelin receptors, suggesting no evidence for species differences. In cells expressing the human apelin receptor, CMF-019 had similar G_αi_ activity to [Pyr^1^]apelin-13 (RE = 4.19), however, for β-arrestin recruitment and internalisation, it was much less potent (RE = 0.01 and 7.19 × 10^−4^ respectively). CMF-019, thus, displayed significant bias compared to the endogenous agonist [Pyr^1^]apelin-13. This bias was towards the G protein signalling pathway, with a bias factor of ∼400 compared to apelin receptor mediated β-arrestin recruitment and ∼6000 compared to β-arrestin mediated receptor internalisation. This is predicted to be beneficial in a therapeutic setting as reduced receptor internalisation would allow for maintained apelin signalling with repeated drug administration. Indeed, our previously published work has shown that MM07, a novel biased peptide agonist, induced reproducible dilatation in the human forearm [4]. This work provided proof of concept for the merits of a biased agonist and our current work has focussed on the investigation of a small molecule agonist with this desirable pharmacological profile. CMF-019 is one of a larger series of apelin agonists based on the benzimidazole scaffold [32] and these additional molecules may also display a similar bias profile.

To our knowledge this is the first report of bias for a small molecule apelin agonist. To date, previous synthetic apelin agonists lack desirable characteristics required of drug-like molecules. E339-3D6, a reported peptidomimetic agonist did exhibit vasoactivity in rat aorta preconstricted with noradrenaline and *in vivo* prevented vasopressin release when injected intracerebroventricularly in water-deprived mice [36]. However, the high molecular weight (1400 Da) suggests it would be an unsuitable drug molecule, particularly for oral dosing. Moreover, it was later shown to be a mixture of polymethylated species from which a number of analogues were subsequently purified [37]. These molecules, displayed low micromolar binding affinities for the apelin receptor compared to CMF-019 which binds in the nanomolar range. ML233, a small molecule of molecular weight of 359 Da, was limited by low solubility in saline at room temperature [38]. Moreover, the structure suggests that it would likely be a pan-assay interference compound (PAINS) and have toxicity, including being a Michael acceptor and having an activated quinone [34]. In contrast to these previously reported compounds, CMF-019 is more drug-like and shows a good physiochemical profile when compared to an oral drug library for Lipinski’s rules (Fig. 8A), Verber’s rules (Fig. 8B) and in a PCA analysis of 15 physiochemical descriptors (Fig. 8C). CMF-019 has one Lipinski’s Rule-of-Five violation (logP), although this is ameliorated by the ionisation of the carboxylic acid at physiological pH, reducing the logD to a more acceptable range (logD = 2.74 at pH7.4). Following the completion of the *in vivo* studies, we detected a mean plasma concentration of ∼25 μM suggesting that CMF-019 is relatively stable with significant compound remaining in the plasma after ten minutes. In contrast, the plasma half-life of [Pyr^1^]apelin-13 in rat has previously been measured to be ∼2 min [4].

### 4.2 CMF-019 affects cardiac action *in vivo*

We examined the effects of CMF-019 *in vivo* when administered to male Sprague–Dawley rats. We used the potassium salt of the compound dissolved in saline at pH9 that showed better solubility than the parent compound. CMF-019 caused a dose dependent increase in dP/dt_MAX,_ an index of increased cardiac contractility. A similar response was observed for [Pyr^1^]apelin-13 and is consistent with its role as an inotropic agent through the apelin receptor. [Pyr^1^]apelin-13 also caused a decrease in LVSP, whereas, CMF-019 caused an increase. The blood pressure response to [Pyr^1^]apelin-13 can be explained by its action as a vasodilatory agent. The fact that CMF-019 did not mirror [Pyr^1^]apelin-13 may simply reflect its limited solubility and a higher concentration might be required to observe an effect on the vasculature. Indeed, it was previously demonstrated that MM07, a novel G protein biased apelin agonist, causes vasodilatation in forearm blood flow studies in human volunteers [4]. Alternatively, the cardiac action of CMF-019 may mask any vasodilator response. However, there is a report that modified apelin-17 peptide fragments biased towards the β-arrestin pathway are more able to induce decreases in blood pressure [39] and this could explain the limited effects of CMF-019 on the vasculature. [Pyr^1^]apelin-13 also increased cardiac output due to an increased stroke volume rather than an increased heart rate. CMF-019 similarly showed a trend to increase stroke volume and cardiac output (with a significant effect observed at 500 nmol) without a change in heart rate. [Pyr^1^]apelin-13 and CMF-019 did not alter the dP/dt_MIN_, suggesting that neither had lusitropic effects. Overall, the responses to CMF-019 showed a similar profile to [Pyr^1^]apelin-13 and are consistent with it acting at the apelin receptor *in vivo*, corroborating the *in vitro* data. Future studies will look at the development of novel compounds to maintain the beneficial bias profile whist improving solubility.

### 4.3 CMF-019 is predicted to bind to a highly conserved region of the apelin receptor

Analysis of the predicted binding region of CMF-019 within the hydrophobic cavity near the SHK region of bound apelin-13 in computational models revealed close proximity to the only reported naturally occurring mutation in the apelin receptor to date. In the zebrafish, *Danio rerio*, a single allele of a recessive mutation, grinch^s608^, leads to a Trp^85^ to Leu^85^ amino acid change in the second transmembrane domain and results in a complete loss of apelin binding. As a consequence of this loss of function, in the most severely affected mutants the heart fails to develop [40]. Interestingly, in the SHK region of apelin-13 in our model is the almost adjacent Tyr^88^ residue. Importantly, both residues have been conserved in humans and all species where apelin receptor sequences have been reported, suggesting CMF-019 binds in a critical region of the receptor. Interestingly, in support of our proposed model, a series of small molecule apelin agonists have been reported [41] that share some structural similarity to CMF-019, having two hydrophobic substituents extending from a heterocyclic core, suggesting that they may bind to the same site of the apelin receptor. It is not yet known if these compounds also display receptor bias.

### 4.4 Conclusions

CMF-019 is the first biased small molecule apelin agonist reported and binds with high affinity to the apelin receptor in human heart homogenate. Binding of CMF-019 was predicted through molecular modelling to occur to a region in the apelin receptor that, by comparison with other species, is highly conserved and is crucial for interaction with the SHK amino acid sequence of apelin-13. In rats, *in vivo*, CMF-019 produced a dose dependent increase in cardiac contractility, corroborating its *in vitro* activity.

Importantly, acute apelin infusion is beneficial in PAH and HF and it is predicted that apelin agonists would display efficacy in treating these conditions. We have previously designed apelin peptide analogues that display bias towards the G protein signalling pathway and have beneficial cardiovascular actions compared with the native peptide in humans *in vivo*. Retention of bias towards G protein signalling over β-arrestin recruitment/internalisation in a small molecule should prove beneficial in a therapeutic setting by reducing receptor downregulation following chronic use, thereby preventing the need for dose escalation or patients becoming refractory to treatment. Consequently, compounds based on the structure of CMF-019 with an improved pharmacokinetic profile could provide a novel and much needed therapy to treat PAH and HF.

## Conflict of interests

None declared.

## Funding

This work was supported by the British Heart Foundation [FS/14/59/31282 to CR]; Wellcome Trust [WT107715/Z/15/Z to APD, REK], Wellcome Trust Programme in Metabolic and Cardiovascular Disease [096822/Z/11/Z to PY]; Medical Research Council [MRC MC PC 14116 to APD, JJM, RCG, PY]; Pulmonary Hypertension Association UK; Cambridge Biomedical Research Centre Biomedical Resources Grant University of Cambridge [099156/Z/12/Z]; Engineering and Physical Sciences Research Council [EP/M506552/1 to CMF]; and the Biomedical Health Research Centre, University of Leeds [CMF, REF].

## Figures and Tables

**Table 1:** Values of ΔLogR and relative effectiveness (RE) for CMF-019 compared to [Pyr^1^]apelin-13 in cAMP, β-arrestin and internalisation assays.

	ΔLogR	RE
β-Arrestin	−1.98 ± 0.18	0.01
Internalisation	−3.14 ± 0.19	7.19 × 10^−4^
cAMP	0.62 ± 0.16	4.19

ΔLogR is Log_10_(τ/K_A_) where τ is a measure of agonist efficacy and K_A_ a measure of functional affinity [35]. n values for each of the assays are as indicated in Fig. 5.

**Table 2:** ΔΔLogR and bias factors (BF, in bold) for CMF-019 compared [Pyr^1^]apelin-13 in cAMP, β-arrestin and internalisation assays.

ΔΔLogR **BF**	β-Arrestin	Internalisation
β-Arrestin	n/a	1.17 ± 0.26
**15**
cAMP	2.60 ± 0.24	3.77 ± 0.25
**398**	**5828**

ΔΔLogR is the difference between ΔLogR values in the different pathways [35].

## References

[1] O’Dowd B.F., Heiber M., Chan A., Heng H.H., Tsui L.C., Kennedy J.L., Shi X., Petronis A., George S.R., Nguyen T. (1993). A human gene that shows identity with the gene encoding the angiotensin receptor is located on chromosome 11. Gene.

[2] Tatemoto K., Hosoya M., Habata Y., Fujii R., Kakegawa T., Zou M.X., Kawamata Y., Fukusumi S., Hinuma S., Kitada C., Kurokawa T., Onda H., Fujino M. (1998). Isolation and characterization of a novel endogenous peptide ligand for the human APJ receptor. Biochem. Biophys. Res. Commun..

[3] Maguire J.J., Kleinz M.J., Pitkin S.L., Davenport A.P. (2009). [Pyr1]apelin-13 identified as the predominant apelin isoform in the human heart: vasoactive mechanisms and inotropic action in disease. Hypertension.

[4] Brame A.L., Maguire J.J., Yang P., Dyson A., Torella R., Cheriyan J., Singer M., Glen R.C., Wilkinson I.B., Davenport A.P. (2015). Design, characterization, and first-in-human study of the vascular actions of a novel biased apelin receptor agonist. Hypertension.

[5] Tatemoto K., Takayama K., Zou M.X., Kumaki I., Zhang W., Kumano K., Fujimiya M. (2001). The novel peptide apelin lowers blood pressure via a nitric oxide-dependent mechanism. Regul. Pept..

[6] Szokodi I., Tavi P., Foldes G., Voutilainen-Myllyla S., Ilves M., Tokola H., Pikkarainen S., Piuhola J., Rysä J., Tóth M., Ruskoaho H. (2002). Apelin, the novel endogenous ligand of the orphan receptor APJ, regulates cardiac contractility. Circ. Res..

[7] Perjés Á., Skoumal R., Tenhunen O., Kónyi A., Simon M., Horváth I.G., Kerkelä R., Ruskoaho H., Szokodi I. (2014). Apelin increases cardiac contractility via protein kinase Cε- and extracellular signal-regulated kinase-dependent mechanisms. PLoS ONE.

[8] Berry M.F., Pirolli T.J., Jayasankar V., Burdick J., Morine K.J., Gardner T.J., Woo Y.J. (2004). Apelin has in vivo inotropic effects on normal and failing hearts. Circulation.

[9] Atluri P., Morine K.J., Liao G.P., Panlilio C.M., Berry M.F., Hsu V.M., Hiesinger W., Cohen J.E., Woo Y.J. (2007). Ischemic heart failure enhances endogenous myocardial apelin and APJ receptor expression. Cell. Mol. Biol. Lett..

[10] Jia Y.X., Pan C.S., Zhang J., Geng B., Zhao J., Gerns H., Yang J., Chang J.K., Tang C.S., Qi Y.F. (2006). Apelin protects myocardial injury induced by isoproterenol in rats. Regul. Pept..

[11] Ashley E.A., Powers J., Chen M., Kundu R., Finsterbach T., Caffarelli A., Deng A., Eichhorn J., Mahajan R., Agrawal R., Greve J., Robbins R., Patterson A.J., Bernstein D., Quertermous T. (2005). The endogenous peptide apelin potently improves cardiac contractility and reduces cardiac loading in vivo. Cardiovasc. Res..

[12] Japp A.G., Cruden N.L., Barnes G., van Gemeren N., Mathews J., Adamson J., Johnston N.R., Denvir M.A., Megson I.L., Flapan A.D., Newby D.E. (2010). Acute cardiovascular effects of apelin in humans: potential role in patients with chronic heart failure. Circulation.

[13] Yang P., Maguire J.J., Davenport A.P. (2015). Apelin, Elabela/Toddler, and biased agonists as novel therapeutic agents in the cardiovascular system. Trends Pharmacol. Sci..

[14] Goetze J.P., Rehfeld J.F., Carlsen J., Videbaek R., Andersen C.B., Boesgaard S., Friis-Hansen L. (2006). Apelin: a new plasma marker of cardiopulmonary disease. Regul. Pept..

[15] Kim J., Kang Y., Kojima Y., Lighthouse J.K., Hu X., Aldred M.A., McLean D.L., Park H., Comhair S.A., Greif D.M., Erzurum S.C., Chun H.J. (2013). An endothelial apelin-FGF link mediated by miR-424 and miR-503 is disrupted in pulmonary arterial hypertension. Nat. Med..

[16] Alastalo T.P., Li M., Perez Vde J., Pham D., Sawada H., Wang J.K., Koskenvuo M., Wang L., Freeman B.A., Chang H.Y., Rabinovitch M. (2011). Disruption of PPARgamma/beta-catenin-mediated regulation of apelin impairs BMP-induced mouse and human pulmonary arterial EC survival. J. Clin. Invest..

[17] Földes G., Horkay F., Szokodi I., Vuolteenaho O., Ilves M., Lindstedt K.A., Mäyränpää M., Sármán B., Seres L., Skoumal R., Lakó-Futó Z., deChâtel R., Ruskoaho H., Tóth M. (2003). Circulating and cardiac levels of apelin, the novel ligand of the orphan receptor APJ, in patients with heart failure. Biochem. Biophys. Res. Commun..

[18] Chong K.S., Gardner R.S., Morton J.J., Ashley E.A., McDonagh T.A. (2006). Plasma concentrations of the novel peptide apelin are decreased in patients with chronic heart failure. Eur. J. Heart Fail..

[19] Francia P., Salvati A., Balla C., De Paolis P., Pagannone E., Borro M., Gentile G., Simmaco M., De Biase L., Volpe M. (2007). Cardiac resynchronization therapy increases plasma levels of the endogenous inotrope apelin. Eur. J. Heart Fail..

[20] Andersen C.U., Markvardsen L.H., Hilberg O., Simonsen U. (2009). Pulmonary apelin levels and effects in rats with hypoxic pulmonary hypertension. Respir. Med..

[21] Barnes G.D., Alam S., Carter G., Pedersen C.M., Lee K.M., Hubbard T.J., Veitch S., Jeong H., White A., Cruden N.L., Huson L., Japp A.G., Newby D.E. (2013). Sustained cardiovascular actions of APJ agonism during renin-angiotensin system activation and in patients with heart failure. Circ. Heart Fail..

[22] Falcao-Pires I., Goncalves N., Henriques-Coelho T., Moreira-Goncalves D., Roncon-Albuquerque R., Leite-Moreira A.F. (2009). Apelin decreases myocardial injury and improves right ventricular function in monocrotaline-induced pulmonary hypertension. Am. J. Physiol. Heart Circ. Physiol..

[23] Koguchi W., Kobayashi N., Takeshima H., Ishikawa M., Sugiyama F., Ishimitsu T. (2012). Cardioprotective effect of apelin-13 on cardiac performance and remodeling in end-stage heart failure. Circ. J..

[24] Wang M., Gupta R.C., Rastogi S., Kohli S., Sabbah M.S., Zhang K., Mohyi P., Hogie M., Fischer Y., Sabbah H.N. (2013). Effects of acute intravenous infusion of apelin on left ventricular function in dogs with advanced heart failure. J. Card. Fail..

[25] Pang H., Han B., Yu T., Zong Z. (2014). Effect of apelin on the cardiac hemodynamics in hypertensive rats with heart failure. Int. J. Mol. Med..

[26] Zhou N., Fan X., Mukhtar M., Fang J., Patel C.A., DuBois G.C., Pomerantz R.J. (2003). Cell-cell fusion and internalization of the CNS-based, HIV-1 co-receptor, APJ. Virology.

[27] Lee D.K., Ferguson S.S., George S.R., O’Dowd B.F. (2010). The fate of the internalized apelin receptor is determined by different isoforms of apelin mediating differential interaction with beta-arrestin. Biochem. Biophys. Res. Commun..

[28] Ceraudo E., Galanth C., Carpentier E., Banegas-Font I., Schonegge A.M., Alvear-Perez R., Iturrioz X., Bouvier M., Llorens-Cortes C. (2014). Biased signaling favoring gi over beta-arrestin promoted by an apelin fragment lacking the C-terminal phenylalanine. J. Biol. Chem..

[29] Chen X., Bai B., Tian Y., Du H., Chen J. (2014). Identification of serine 348 on the apelin receptor as a novel regulatory phosphorylation site in apelin-13-induced G protein-independent biased signaling. J. Biol. Chem..

[30] Jones G., Willett P., Glen R.C. (1995). Molecular recognition of receptor sites using a genetic algorithm with a description of desolvation. J. Mol. Biol..

[31] Jones G., Willett P., Glen R.C., Leach A.R., Taylor R. (1997). Development and validation of a genetic algorithm for flexible docking. J. Mol. Biol..

[32] S. Hachtel, P. Wohlfart, J. Weston, M. Müller, E. Defossa, K. Mertsch, J. Weng, Robert A. Binnie, F. Abdul-latif, W.J. Bock, A. Walser, Benzoimidazole-carboxylic acid amide derivatives as APJ receptor modulators. Google Patents, 2014, WO2014044738 A1.

[33] Pacher P., Nagayama T., Mukhopadhyay P., Batkai S., Kass D.A. (2008). Measurement of cardiac function using pressure-volume conductance catheter technique in mice and rats. Nat. Protoc..

[34] Lagorce D., Sperandio O., Baell J.B., Miteva M.A., Villoutreix B.O. (2015). FAF-Drugs3: a web server for compound property calculation and chemical library design. Nucleic Acids Res..

[35] van der Westhuizen E.T., Breton B., Christopoulos A., Bouvier M. (2014). Quantification of ligand bias for clinically relevant beta2-adrenergic receptor ligands: implications for drug taxonomy. Mol. Pharmacol..

[36] Iturrioz X., Alvear-Perez R., De Mota N., Franchet C., Guillier F., Leroux V., Dabire H., Le Jouan M., Chabane H., Gerbier R., Bonnet D., Berdeaux A., Maigret B., Galzi J.L., Hibert M., Llorens-Cortes C. (2010). Identification and pharmacological properties of E339-3D6, the first nonpeptidic apelin receptor agonist. FASEB J..

[37] Margathe J.F., Iturrioz X., Alvear-Perez R., Marsol C., Riche S., Chabane H., Tounsi N., Kuhry M., Heissler D., Hibert M., Llorens-Cortes C., Bonnet D. (2014). Structure-activity relationship studies toward the discovery of selective apelin receptor agonists. J. Med. Chem..

[38] P. Khan, P.R. Maloney, M. Hedrick, P. Gosalia, M. Milewski, L. Li, G.P. Roth, E. Sergienko, E. Suyama, E. Sugarman, K. Nguyen, A. Mehta, S. Vasile, Y. Su, D. Stonich, H. Nguyen, F.Y. Zeng, A.M. Novo, M. Vicchiarelli, J. Diwan, T.D.Y. Chung, A.B. Pinkerton, L.H. Smith, Functional Agonists of the Apelin (APJ) Receptor. Probe Reports from the NIH Molecular Libraries Program 2010.

[39] El Messari S., Iturrioz X., Fassot C., De Mota N., Roesch D., Llorens-Cortes C. (2004). Functional dissociation of apelin receptor signaling and endocytosis: implications for the effects of apelin on arterial blood pressure. J. Neurochem..

[40] Scott I.C., Masri B., D’Amico L.A., Jin S.W., Jungblut B., Wehman A.M., Baier H., Audigier Y., Stainier D.Y. (2007). The g protein-coupled receptor agtrl1b regulates early development of myocardial progenitors. Dev. Cell.

[41] A.B. Pinkerton, L.H. Smith, Agonists of the apelin receptor and methods of use thereof. Google Patents 2015, WO2015184011 A3.

